# Implementing and evaluating a comprehensive simulation-based program to enhance nursing students’ coping with death, burnout, and communication skills

**DOI:** 10.3389/fpsyg.2026.1828226

**Published:** 2026-06-30

**Authors:** Miguel Ángel Martín-Parrilla, Noelia Durán-Gómez, Marta Nadal-Delgado, Casimiro Fermín López-Jurado, Patricia Palomo-López, Macarena C. Cáceres

**Affiliations:** 1 Departamento de Enfermería, Centro Universitario de Plasencia, Universidad de Extremadura, Plasencia, Spain; 2 Grupo de Investigación Traslacional Biomédica y Sociosanitaria (CTS064), Badajoz, Spain; 3 Instituto Universitario de Investigación Biosanitaria de Extremadura (INUBE), Badajoz, Spain; 4 Departamento de Enfermería, Facultad de Medicina y Ciencias de la Salud, Universidad de Extremadura, Badajoz, Spain; 5 Hospital Universitario de Badajoz, Asociación Oncológica Extremeña (AOEX), Badajoz, Spain

**Keywords:** burnout, communication skill, coping with death, emotional intelligence, fear of death, mental health nursing, nursing education research, satisfaction

## Abstract

**Background/objectives:**

Current reforms in higher education emphasize the need for active student engagement as a key element of competency-based learning. Within nursing education, simulation-based learning experiences (SBLE) provide an effective pedagogical strategy to integrate practical and emotional skills, especially in palliative care contexts. This study aimed to implement and evaluate a comprehensive simulation-based educational program designed to enhance nursing students’ coping with death, reduce burnout, and improve communication skills, thereby fostering compassionate and emotionally competent care.

**Methods:**

A program evaluation design with a one-group pretest–posttest educational intervention was employed in accordance with the TREND statement. Participants were second-year undergraduate nursing students. Baseline measures assessed fear of death and emotional intelligence. The intervention included pre-briefing sessions on death, burnout, and communication with terminal patients, followed by simulation and debriefing phases. Outcomes included students’ coping capacity with death, burnout levels (emotional exhaustion, cynicism, and self-efficacy), communication skills in clinical interactions, and satisfaction with SBLE.

**Results:**

Before the intervention, students exhibited moderate to high levels of fear of death, particularly concerning others’ death, and adequate emotional intelligence levels. Post-intervention results showed statistically significant improvements in coping with death (*p* < 0.001), reductions in emotional exhaustion and cynicism, and increased self-efficacy. Communication skills improved notably, supported by both instructor evaluations and student self-assessments. Overall satisfaction with the SBLE was high.

**Conclusion:**

Simulation-based learning effectively enhances nursing students’ emotional preparedness, coping capacity, and communication competence while reducing burnout. These outcomes support the integration of SBLE into nursing curricula to foster compassionate, resilient, and skilled professionals in end-of-life and emotionally demanding care settings.

## 1 Introduction

Ongoing calls for reform in university education emphasize the need to prioritize active student engagement (Micallef, 2025; UNESCO, 2019). This is especially relevant in competency-based education, where practical learning must be closely integrated with theoretical knowledge. In this context, simulations emerge as an innovative pedagogical method, engaging learners through various sensory modalities (Elendu et al., 2024; Eyikara and Baykara, 2017).

Simulation-based learning experiences (SBLE) are designed to replicate real-world processes with sufficient fidelity to achieve specific educational objectives, providing learners with immersive clinical scenarios. This method improves students’ confidence and competence, particularly in managing complex situations such as end-of-life care (Alrashidi et al., 2023). Integrating SBLE into nursing curricula is essential for preparing future nurses to effectively navigate the challenges of healthcare environments (Chabrera et al., 2024). The introduction of Benner’s Novice to Expert Model provides a valuable framework for understanding how SBLE supports the progressive development of nursing students (Graf et al., 2020; Lamichhane, 2025). According to Benner (1984), nurses advance through five stages of competency: novice, advanced beginner, competent, proficient, and expert. Simulation allows students to develop essential skills like critical thinking and teamwork in a safe, structured environment. In this way, SBLE facilitates the development of confidence and competence in managing complex healthcare situations, supporting students’ progression along Benner’s continuum of clinical experience (Benner, 1984; Healy and Lonergan, 2024).

Nurses often care for terminally ill patients, which can increase awareness of mortality and lead to emotional distress (De Brasi et al., 2021; Kiarie et al., 2026). Early exposure to death during nursing education reveals inadequacies in preparing students to effectively cope with the emotional and psychological dimensions of such situations (Wu et al., 2023). The emotional toll experienced by nursing students underscores the pressing need for enhanced training in emotional and psychological well-being (Haver et al., 2025; Reverté-Villarroya et al., 2021; Szczupakowska et al., 2021). Managing the dying process is vital, particularly since traditional training focuses more on curing than on end-of-life care (Sutherland, 2019). Simulation-based learning is integral to the palliative care curriculum, providing a realistic and interactive platform for students to develop critical skills. Involving clinical experts as facilitators is essential, as they offer valuable insights and real-world perspectives. Additionally, considering students’ prior experiences with death and dying is vital, as these experiences can significantly impact their competencies in palliative and end-of-life care, either positively or negatively (Skedsmo et al., 2023; Yoong et al., 2024).

Fear of death has been empirically linked to emotional intelligence (EI) (Fernández-Martínez et al., 2021; Martínez-Jabares et al., 2025), and deficiencies in coping self-efficacy can precipitate burnout among nursing professionals (Lin et al., 2022). Burnout, characterized by negative attitudes and feelings towards one’s professional role in response to chronic work stress, is particularly pervasive in healthcare contexts and contributes to workforce shortages (Arian et al., 2023). Likewise, it has been shown to be a problem among undergraduate students (Gómez-Urquiza et al., 2023), with the latest evidence indicating a prevalence of 46% in this future professional profile (Arian et al., 2023). Coping self-efficacy helps protect against burnout, highlighting the importance of training related to death and dying (Ogińska-Bulik and Michalska, 2021; Zheng et al., 2022). The integration of these concepts into nursing education curricula is imperative, as burnout directly compromises the quality of patient care (Küçük Yüceyurt and Kaya, 2026; Turan et al., 2019).

Improving nurses’ emotional readiness benefits both their well-being and the quality of patient care (Molina-Mula and Gallo-Estrada, 2020; Toluk et al., 2025). Proficiency in navigating the challenges associated with death is essential for mitigating burnout, as effective coping strategies entail the management of psychological stressors (Alodhialah et al., 2024; Zheng et al., 2018). Effective communication skills play a pivotal role in patient care, facilitating adaptation, preserving autonomy, and alleviating anxiety (Kwame and Petrucka, 2021; Ominyi and Alabi, 2025). Despite its importance, emotional and psychological training in healthcare remains under-researched (Wiedermann et al., 2023). Consequently, there exists a pressing need for further research endeavours aimed at augmenting nursing competencies during university education (Martín-Parrilla et al., 2025a).

Effective communication in end-of-life care is considered one of the most challenging non-technical competencies for undergraduate nursing students, particularly when interacting with terminally ill patients and their families (Taheri-Ezbarami et al., 2024). Recent evidence suggests that communication training within SBLE may be especially effective because it allows students to practice difficult conversations in psychologically safe and clinically realistic environments. In this context, structured pre-briefing plays a relevant role by clarifying communication objectives, reducing anticipatory anxiety, and preparing students emotionally before simulation exposure (Madsgaard and Svellingen, 2025). However, communication skill acquisition appears to be more strongly associated with the experiential component of SBLE itself, as simulation enables active interaction, immediate feedback, reflective learning, and repeated practice of complex communicative behaviours (Gogollari et al., 2026).

The aim of this study was to implement and evaluate a comprehensive simulation-based educational program designed to enhance nursing students’ capacities to cope with death, prevent burnout, and strengthen communication skills in end-of-life care contexts. This program evaluation aimed to assess the educational effectiveness, feasibility, and perceived value of the integrated model within undergraduate nursing education.

Based on the existing evidence regarding simulation-based learning in nursing education, we hypothesized that participation in the comprehensive SBLE program would significantly improve nursing students’ coping with death and communication skills, reduce burnout levels, and generate high levels of satisfaction with the educational experience after the intervention compared with baseline measurements.

## 2 Materials and methods

### 2.1 Design

This study followed a program evaluation approach using a one-group pretest–post-test educational intervention design, conducted in accordance with the TREND statement for transparent reporting of non-randomized designs (Des Jarlais et al., 2004). The continued relevance of the TREND statement is evidenced by its ongoing use in recent nursing and health professions education research involving non-randomized educational interventions (Creighton et al., 2025).

### 2.2 Study population and setting

The sample consisted of 100 undergraduate students enrolled in the Nursing Degree program at the University of Extremadura (Spain) during the academic course 2022/2023. Data were specifically collected from 1 April 2023 to 6 May 2023. As part of the program evaluation framework, all eligible students were invited to participate, ensuring that the data reflected the experience of the full cohort exposed to the intervention.

The inclusion criteria for the study cohort comprised enrolment of second-year Nursing students at the University of Extremadura, contingent upon the signing of informed consent. According to the university’s nursing curriculum, second-year students have not yet had any clinical or hospital-based experience. In contrast, the exclusion criteria encompassed the disqualification of individuals with prior experience in hospital settings, partial participation in the training, recent or pathological grief, current or recent receipt of psychological care related to loss or mourning, as well as those presenting very low levels of emotional intelligence, in order to minimize potential emotional distress during exposure to end-of-life simulation scenarios and to ensure participant safety during the intervention. These exclusions were implemented to ensure the homogeneity of the study group and to mitigate the influence of external variables on the obtained results.

### 2.3 Program description

**Figure 1:**
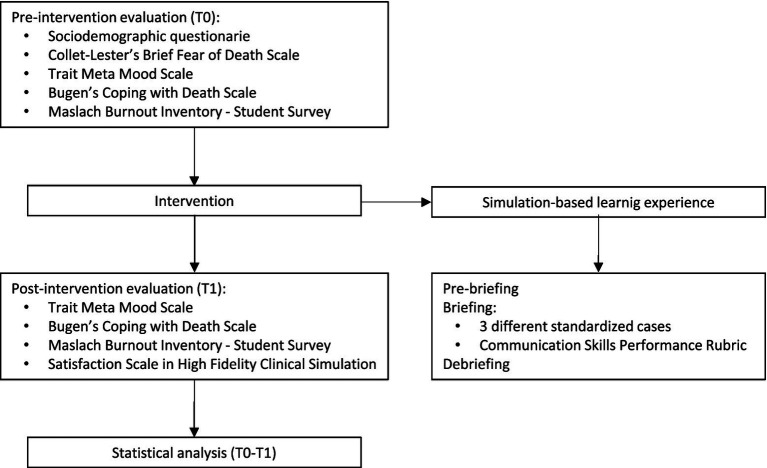
Data collection procedure flowchart.

The procedure of the present study is shown in Figure 1.

The decision was made to comply with the Healthcare Simulation Standards of Best Practice™ Facilitation, a framework for ensuring high-quality healthcare simulation education and training (Persico et al., 2021). These standards continue to guide the design and implementation of contemporary simulation-based educational interventions and have been applied in recent nursing education research to ensure methodological rigor, learner engagement, and educational quality (Scott, 2026).

#### 2.3.1 Pre-briefing

The format consisted of the following itinerary: (i) the meaning of death, (ii) evolution of the death process, (iii) what death means for everyone: experiential exercise, (iv) the sociology of death, (v) how to communicate with a terminal patient: counselling, and (vi) how the death of others affects us as future health professionals: burnout.

The instructors provided the students with the following preparatory material: (i) an explanation of the SBLE goals, objectives, and format; (ii) materials that would be necessary for the simulation, and (iii) an electronic book to help them prepare for the clinical cases with some samples.

During the pre-briefing, faculty facilitators explained all about the potential patients by giving the students their medical histories. Learners had the opportunity to clarify their doubts prior to the SBLE. Trained actors were also present.

#### 2.3.2 Simulation design and procedures: briefing

The SBLE lasted approximately 50 min per group, with a total of three cases addressed in the simulation exercises. The simulation scenarios were designed to represent realistic and progressively complex end-of-life care situations commonly encountered in clinical practice. Across the three cases, students were exposed to patients at different stages of advanced illness, requiring adaptation to varying levels of emotional distress, symptom burden, and communication needs.

One scenario involved a patient with advanced chronic deterioration admitted to palliative care, presenting significant emotional distress related to uncertainty and fear of disease progression and death. Students were required to provide psychological support and establish therapeutic communication in the context of high emotional vulnerability.

A second scenario focused on a young adult with advanced disease progression and severe clinical complications, characterized by heightened anxiety and uncertainty regarding prognosis and transition to end-of-life care. The emphasis was placed on managing emotionally charged communication while supporting coping and understanding of the clinical situation.

The third scenario represented a younger patient with metastatic oncological disease and advanced functional decline, requiring intensive symptom management and end-of-life care planning. Students had to integrate communication skills with emotional support strategies while ensuring respect for patient values, dignity, and preferences.

As part of the simulation, all learners portrayed themselves as second-year Nursing Degree students and were accompanied by two trained actors: one real nurse actor in the role of the nurse and the other as the patient. Some students actively participated, while observers took notes and assessed their peers in the subsequent phase of the simulation process. However, no stratified analysis was conducted according to participation role (active participant vs. observer), as all students were exposed to the same simulation scenarios and debriefing process as part of a unified educational intervention. The performances were recorded.

An instructor was present at all times to evaluate the simulation process.

#### 2.3.3 Debriefing

Immediately following the simulation, all the groups that participated in the SBLE started the debriefing. The framework employed for the debriefing process was subdivided into three components: simulation visualization, observer discussion, and instructor feedback (Reierson et al., 2017).

### 2.4 Instrument and measures

Sociodemographic questionnaire: It is tailored specifically for this study. It encompasses inquiries about the students’ age, gender, and academic achievements, including grades and clinical experiences. This questionnaire is included in the supplementary material. The collection of sociodemographic and academic variables is a standard methodological procedure in health and educational research, allowing researchers to characterize study samples and identify potential factors influencing educational outcomes. Recent nursing education studies continue to employ tailored sociodemographic questionnaires to contextualize participant characteristics and support the interpretation of intervention effects (Galan-Lominchar et al., 2024).

The Collet-Lester’s Brief Fear of Death Scale (BFDS): This segment aims to gauge the students’ fears surrounding death and the dying process, both for themselves and for others. The BFDS consists of four distinct subscales, each comprise of seven items, adding up to a total of 28 items. Respondents were asked to rate their feelings on a Likert-type scale, ranging from 1 (not at all) to 5 (a lot). The total score and scores for each subscale were calculated to measure the degree of fear related to death and the dying process. Internal consistency of the scale was strong, with Cronbach’s alpha coefficients of 0.91 for fear of one’s own death, 0.92 for fear of one’s own dying process, 0.88 for fear of others’ death, and 0.92 for fear of others’ dying process, indicating high reliability across all dimensions. Moreover, the validity of the scale is also supported by these results, confirming its robustness for assessing this construct in the studied population (Mondragon-Sanchez et al., 2020). More recent studies continue to support the validity and applicability of the instrument in health sciences education and cross-cultural contexts, including psychometric validation studies conducted in occupational therapy students and European populations (Gutiérrez-Sánchez et al., 2024).

The Trait Meta Mood Scale (TMMS-24): Derived from the Trait Meta-Mood Scale developed by the Salovey and Mayer research group, this scale delves into the participants’ emotional intelligence. It assesses their ability to recognize and regulate their own emotions. The TMMS-24 focuses on three crucial dimensions of emotional intelligence: emotional attention, emotional clarity, and emotional repair. In the context of standard values for adequate attention, adequate clarity, and adequate repair, differences based on gender are observed according to total scores. For adequate attention, the optimal scores for men range from 22 to 32, while for women, they range from 25 to 35. Regarding adequate clarity, the standard values are between 26 and 35 for men and between 24 and 34 for women. Lastly, adequate repair is achieved with scores ranging from 24 to 35 for men and from 24 to 34 for women. The instrument demonstrated good internal consistency across all three subscales, with Cronbach’s alpha coefficients of 0.82 for emotional attention, 0.84 for emotional clarity, and 0.85 for emotional repair, indicating strong reliability. Additionally, the validity of the instrument has been statistically demonstrated through confirmatory factor analysis and significant correlations with related constructs, supporting its construct validity (Gonzalez et al., 2020). Recent studies continue to employ and validate the TMMS-24 in university and healthcare student populations, confirming its reliability and construct validity for assessing EI dimensions in educational settings (Fernández-Berrocal et al., 2025).

The Bugen’s Coping with Death Scale (CDS): This questionnaire seeks to evaluate the students’ capacity to manage death and their preparedness for dealing with it. Comprising 30 items, it employs a Likert-type scale ranging from 1 (strongly disagree) to 7 (strongly agree). The total score for competence in coping with death is derived by summing all items, with a few items (1, 13, and 24) being inverted. The total score could range between 30 and 210, and the psychometric properties of this scale were explored in the research, with Cronbach’s alpha coefficient reported at 0.89, reflecting solid internal consistency. In addition, construct validity was supported by empirical evidence derived from factor analytic procedures and meaningful associations with conceptually relevant variables (Galiana and Oliver, 2017). More recent studies continue to support its applicability in palliative care and nursing education contexts, particularly in research exploring emotional preparedness and coping self-efficacy related to death and dying (Esteban-Burgos et al., 2024).

The Maslach Burnout Inventory-Student Survey (MBI-SS): This questionnaire is utilized to assess academic burnout in students. It probes into their feelings of physical and mental exhaustion, cynicism, and self-doubt concerning their academic abilities. The MBI-SS features three distinct subscales, each comprising a different number of items. Respondents use a 7-point frequency scale, ranging from 0 (never) to 6 (always), to express their experiences (Hederich-Martínez and Caballero-Domínguez, 2016). The instrument demonstrated acceptable to good internal consistency, with Cronbach’s alpha coefficients of 0.77 for exhaustion, 0.72 for cynicism, and 0.82 for reduced efficacy. Furthermore, its construct validity has been empirically supported through confirmatory factor analysis and observed relationships with related psychological constructs (Hederich-Martínez and Caballero-Domínguez, 2016). Recent studies continue to employ the MBI-SS extensively in university student populations supporting its ongoing relevance and psychometric adequacy for evaluating academic burnout and emotional exhaustion in higher education contexts (Chong et al., 2025).

The Satisfaction Scale in High Fidelity Clinical Simulation (ESSAF): This self-administered questionnaire consists of 33 items and employed a 5-point Likert-type response scale. Students are asked to rate their level of agreement or disagreement with the statements, covering their satisfaction with high-fidelity clinical simulations. The instrument showed good internal consistency, with a Cronbach’s alpha coefficient of 0.86. In addition, construct validity was supported through statistical analyses, including factor structure confirmation and its alignment with theoretically expected dimensions of student satisfaction (Alconero-Camarero et al., 2016). In the last few years, the instrument has continued to be employed in simulation-based nursing education research, supporting its continued utility for assessing satisfaction, perceived learning, and educational experience associated with simulation methodologies in healthcare training contexts (Jiménez-Álvarez et al., 2024).

The Communication Skills Performance Rubric (CSPR): This tool assesses communication abilities in clinical simulation using 15 items and a five-level Likert scale. The “Simulation Performance” dimension evaluates communication and teamwork competencies (scored from 11 to 55). The “Debriefing Evaluation” dimension assesses participation in the post-simulation review (scored from 4 to 20). Both dimensions provide a continuous interpretation of performance. The instrument demonstrated strong psychometric properties. Cronbach’s alpha coefficients were 0.93 for the Simulation Performance dimension and 0.80 for the Debriefing Evaluation dimension. Moreover, evidence of construct validity has been established through statistical analyses, including factor structure confirmation and convergence with theoretically related measures (Juliá-Sanchis et al., 2023). This rubric was not included in the questionnaires as it was employed by the instructor for the debriefing process.

All questionnaires are open access except for the CSPR, for which permission was requested and received from the authors.

### 2.5 Statistical analysis

For data processing, we utilized version 2.3 of the jamovi statistical software (The Jamovi Project, 2022). Specifically, we employed descriptive analysis and a Paired Samples T-Test to analyse the collected data. For all analyses, the *α*-level was set at *p* < 0.05.

### 2.6 Ethical consideration

The Bioethics and Biosafety Commission of the University of Extremadura confirmed that the study complied to essential ethical standards (reg. Number: 50//2023). This encompassed ensuring the voluntary nature of participation in the project, with all participants signing informed consent and guaranteeing anonymity.

## 3 Results

The sample consisted of a group of a hundred (*n* = 100) undergraduate students. There were 76 women (76%) and 24 men (24%). The average age was 21.3 (SD = 4.44). The remaining sociodemographic and academic characteristics of the sample are presented in Table 1.

**Table 1:** Sociodemographic and academic characteristics of the sample.

Variable	Category	n	Porcentaje
Access to nursing degree	EBAU	67	67.0
	Vocational Training (FP)	26	26.0
	Over 25 admission route	5	5.0
	Other degree holders	2	2.0
Outstanding subjects from previous year	No	87	87.0
	Yes	13	13.0
Scholarship status	No	38	38.0
	Yes	62	62.0
Interest at entry into degree	Very high	56	56.0
	High	37	37.0
	Medium	7	7.0
Current interest in degree	Very high	49	49.0
	High	44	44.0
	Medium	7	7.0
Interest in this training (SBLE)	Very high	44	44.0
	High	46	46.0
	Medium	10	10.0
Study–work balance	No	89	89.0
	Yes	11	11.0
Additional caregiving responsibilities	No	91	91.0
	Yes	9	9.0
Stress/relaxation activities	No	59	59.0
	Yes	41	41.0
Recent bereavement process	No	80	80.0
	Yes	20	20.0
Psychological support	No	71	71.0
	Yes	29	29.0
Chronic illness	No	88	88.0
	Yes	12	12.0

### 3.1 Pre intervention analysis

Prior to the intervention, it was necessary to analyse the fear of death and the EI, as shown in Table 1.

The sample of men and women in this study had levels of fear of death classified between normal and high, and an adequate average EI, and so were able to participate in the project. Regarding fear of death, the high level of fear of the death of others stands out above all. Death is worse assimilated than the process itself.

### 3.2 Pre-post intervention analysis

Table 2 shows the results in terms of enhancement on coping with death abilities and burnout reduction in the sample after the intervention.

**Table 2:** Fear of death and emotional intelligence.

Variable	Female (x̄±SD) *n* = 76	Male (x̄±SD) *n* = 24	Total (x̄±SD) *n* = 100
Collet-Lester’s brief fear of death scale			
Fear of one’s own death	21.8 ± 6.59	18.2 ± 4.87	21.0 ± 6.39
Fear of the one’s dying	23.8 ± 5.38	20.8 ± 5,81	23.1 ± 5.61
Fear of death of others	26.9 ± 4.73	25.2 ± 3.99	26.5 ± 4.60
Fear of the dying of others	23.7 ± 5.22	21.2 ± 3.08	23.1 ± 4.89
Trait meta mood scale-24 items			
Emotional attention	30.0 ± 5.33	29.4 ± 4.01	
Emotional clarity	26.8 ± 5.07	27.4 ± 5.44	

The statistically significant improvements observed in coping with death and burnout indicators suggest not only measurable changes but also potential clinical relevance. The 24.5-point increase in CDS reflects a substantial enhancement in students’ perceived competence and emotional preparedness to deal with death, which is a critical skill in palliative and end-of-life care settings. The 1.2-point reduction in exhaustion and 0.5-point decrease in cynicism on the MBI-SS, although modest in numerical terms, represent meaningful progress in mitigating early signs of burnout among healthcare students. Moreover, the 0.7-point increase in reduced efficacy is indicative of strengthened confidence in clinical performance.

### 3.3 Post intervention analysis

The results relating to the students’ satisfaction in participating in SBLE are shown in Table 3.

**Table 3:** Paired samples *t*-test of coping with death and burnout levels.

*n* = 100	Pre-intervention (x̄±SD)	Post-intervention (x̄±SD)	Mean difference (95% CI)	*p*-value	Effect size (Cohen’s d)
Bugen’s coping with death scale					
Coping with death	107.7 ± 19.31	132.2 ± 19.34	+24.50 (19.7, 29.4)	< 0.001	1.0
Maslach burnout inventory-student survey					
Exhaustion	3.6 ± 1.20	2.4 ± 1.10	−1.20 (−1.3, −1.1)	< 0.001	−2.0
Cynicism	2.0 ± 1.22	1.5 ± 1.00	−0.50 (−0.6, −0.4)	< 0.001	−1.1
Reduced efficacy	3.0 ± 0.80	3.7 ± 0.90	+0.70 (0.6, 0.8)	< 0.001	1.5

**Figure 2:**
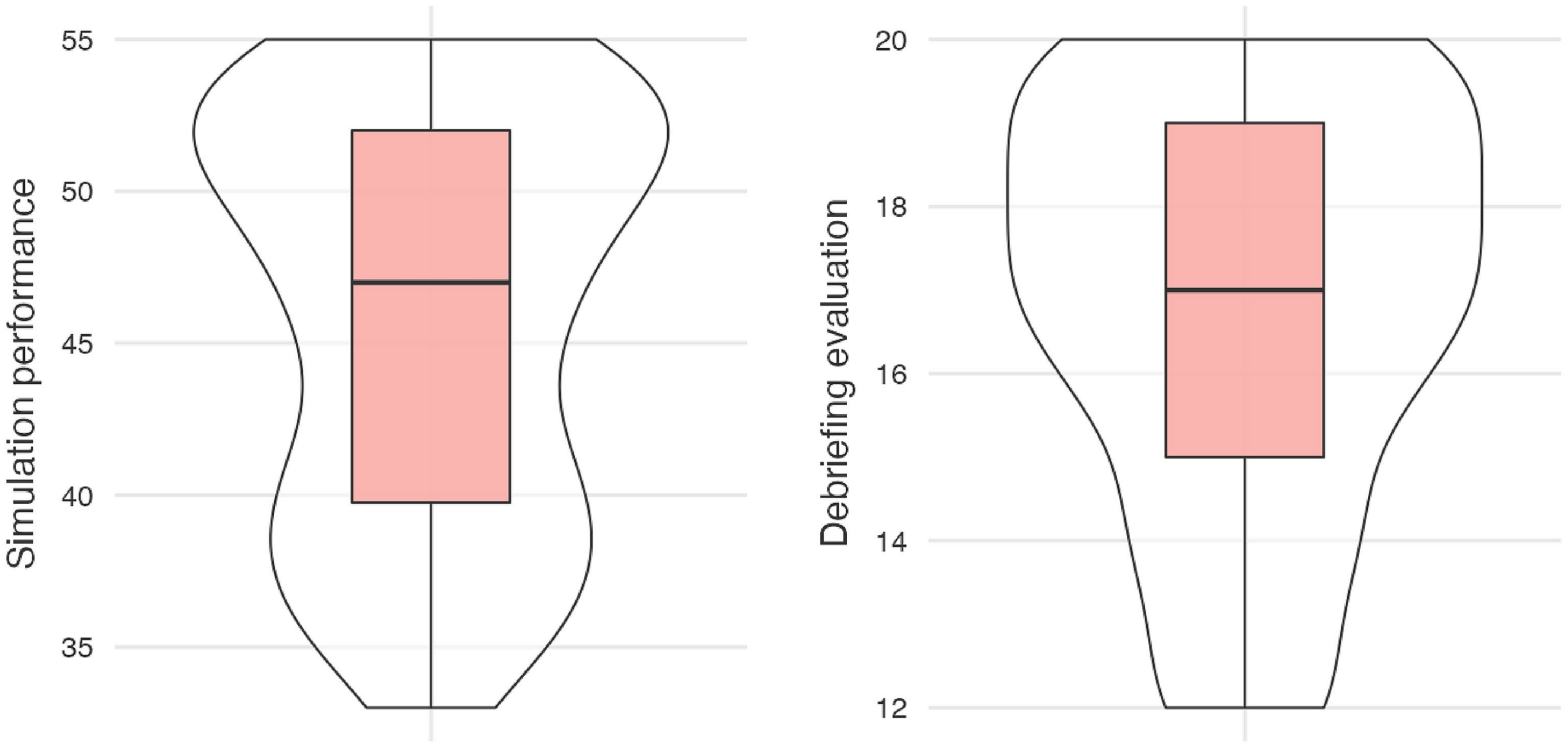
Results of the communication skills performance rubric measured during briefing and debriefing.

The results obtained with the CSPR tool are as described in Figure 2. The dataset provides a glimpse into participants’ experiences, in the dimensions of simulation performance and debriefing evaluation. In terms of simulation performance, the mean score stands at 45.9 (SD = 6.8). Scores ranged from 33 to 55, underscoring the variance and distinct proficiency levels among participants (see Table 4).

**Table 4:** Results of the satisfaction scale in high fidelity clinical simulation.

*n* = 100	Mean	SD
Facilities and equipment were real	4.36	0.772
Objectives were clear cases	4.59	0.767
Cases recreated real situations	4.66	0.670
Timing for each simulation case was adequate	4.66	0.590
The degree of case difficulty was appropriate to my knowledge	4.35	0.880
I felt comfortable and respected during the sessions	4.48	0.870
Clinical simulation is useful to assess a patient’s clinical situation	4.51	0.772
Simulation practices help you to learn to avoid mistakes	4.57	0.655
Simulation has helped me to set priorities for action	4.35	0.880
Simulation has improved my ability to provide my patients with care	4.51	0.718
Simulation has made me think about my upcoming clinical practice	4.52	0.893
Simulation improves communication and teamwork	4.59	0.726
Simulation has made me more aware/concerned about clinical practice	3.53	1.291
Simulation is beneficial to relate theory to practice	4.52	0.785
Simulation allows us to plan patient care effectively	4.12	0.988
I have improved my technical skills	3.83	1.138
I have reinforced my critical thinking and decision-making	4.29	0.832
Simulation has helped me to assess a patient’s condition	4.45	0.770
This experience has helped me prioritize care	4.26	0.906
Simulation promotes self-confidence	4.32	0.920
I have improved communication with the team	4.48	0.858
I have improved communication with the family	4.05	1.132
I have improved communication with the patient	4.58	0.768
This type of practice has increased my confidence	4.04	0.898
I was thrown off balance during some of the cases	3.18	1.382
Interaction with simulation has improved my clinical competence	4.14	0.888
The teacher gave constructive feedback after each session	4.67	0.805
Debriefing has helped me reflect on the cases	4.64	0.674
Debriefing at the end of the session has helped me to correct mistakes	4.59	0.767
I found out about the theoretical side of the cases	4.38	0.850
I have learned from the mistakes I made during the simulation	4.62	0.763
Practicality	4.59	0.780
Overall satisfaction with the sessions	4.62	0.736

Shifting the focus to debriefing evaluation, the mean score is 16.9 (SD = 2.5), representing the average assessment of the debriefing session by participants. The data ranged between the values of 12 and 20.

Regarding communicative competencies, a correlation study was conducted between students’ perceived improvements in communication with teams, families, and patients, and those evaluated through rubric-based assessments by instructors during simulated exercises.

**Figure 3:**
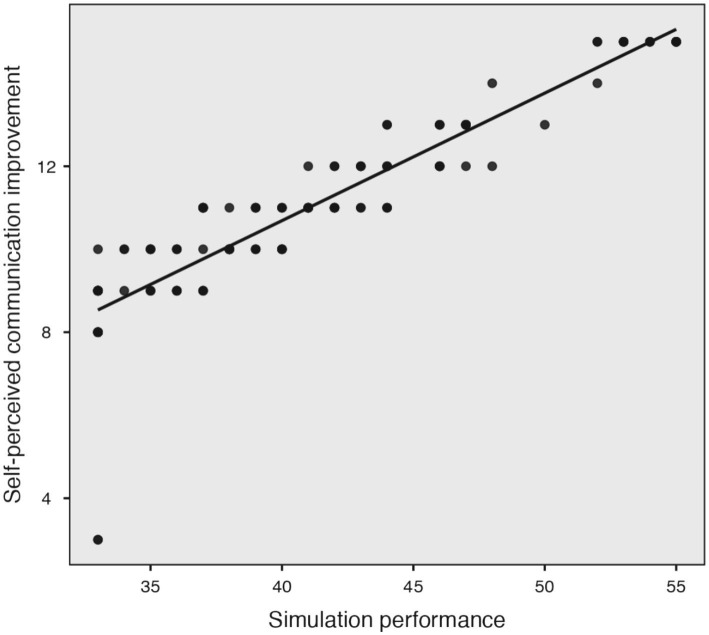
Scatter plot of self-perceived communication by students and performance in simulation evaluated by the instructor.

The Figure 3 shows the relationship between self-perception items of communication improvement from ESSAF and communicative competencies assessed by instructors during briefing development was studied. Items 21–23 of ESSAF were compared with items 1–11 of CSPR for this purpose. The objective of this analysis is to demonstrate whether there is improvement in communicative competencies among students from both student and instructor perspectives.

The mean total score for the three ESSAF items was 11.0 ± 2.23. Pearson’s correlation analysis yielded a coefficient of 0.915 (*p* < 0.001), with a coefficient of determination (R^2^) of 0.84. As shown, self-perceived improvement in communicative competencies correlates with instructors’ perceptions during proficiency evaluations.

Regarding debriefing, a correlation analysis was conducted to examine students’ perception of how beneficial debriefing is perceived to be compared to instructor-collected data during this stage.

**Figure 4:**
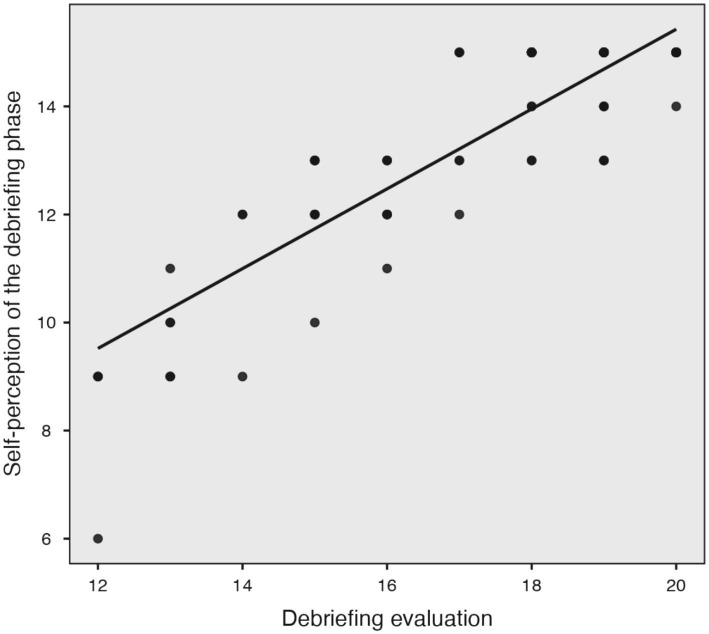
Scatter plot of the debriefing phase self-perceived by the students and the simulation performance evaluated by the instructor.

The Figure 4 describes the relationship between debriefing assessment, evaluated by the instructor in the CSPR, and self-perception items of the stage in the respective items of the ESSAF was examined. Specifically, items 27 to 29 of the ESSAF were compared with items 12 to 15 of the CSPR. The aim of this analysis was to demonstrate the functionality of debriefing from both student and instructor perspectives.

The mean of the sum of the three items on the ESSAF was 14.0 ± 1.85. Pearson correlation analysis revealed a coefficient of 0.889 (p < 0.001), with a corresponding R^2^ of 0.79. As observed, self-perception of improvement through debriefing correlates with the instructor’s perception when conducting competency assessments.

## 4 Discussion

### 4.1 Pre intervention analysis

First, it was necessary to analyse by a cross-sectional design whether the fear of death and EI are indeed a concern among nursing students. This was required to ascertain if the sample was suitable to initiate the learning process. According to our findings, similar to previous studies, which reported moderately high levels of fear of death among students (Fernández-Martínez et al., 2021; Hernández-Gamboa et al., 2024). The overall mean of respondents across the different subscales provides a measure of the intensity of fear of death in the studied sample. This fact is consistent with the contemporary understanding of fear of death as a multidimensional construct that can be influenced by a variety of factors, including cultural background, personal experiences, and philosophical beliefs (Hernández et al., 2024; Kastenbaum, 2024; Menzies et al., 2018).

Following this, what is expected before carrying out the pre-briefing is that the sample has a suitable EI for attending to said intervention. Our results, the sample is within the limits to be considered adequate in all cases, according to the specified standard values for this objective (Harrafa et al., 2024; Toscano-Hermoso et al., 2020).

Nursing students face emotionally challenging situations, making it crucial to understand their emotional attention (Cleary et al., 2018; Lubbe et al., 2026). This ability influences the quality of patient care and their emotional well-being. Effective emotional management enhances empathy and effectiveness in care, reducing stress and burnout (Karimi et al., 2021; El Naggar et al., 2025). Additionally, understanding the level of emotional clarity and repair can guide professional and educational development, implementing specific programs to strengthen these skills and better prepare students for the emotional challenges of their careers (Lewis et al., 2017; Shengyao et al., 2024). This contributes to the overall improvement of the healthcare system, enhancing not only nurse–patient relationships but also nurse–nurse collaboration (Al-Hamdan et al., 2021; Chatzidimitriou et al., 2025).

### 4.2 Pre-post intervention analysis

Our findings suggest that the pedagogical methodology proposed in our study shows promising results in terms of reducing coping with death and burnout levels.

Regarding coping with death, an increase in this capacity is perceived after SBLE. Self-efficacy in the face of death is undoubtedly improved, with regard to the problems previously reported. This suggests that structured experiential learning, such as simulation, may help bridge the emotional and cognitive gaps that traditional curricula leave unaddressed. Other authors conclude that new graduate nurses are at a disadvantage in terms of death self-efficacy, are more anxious about death, and are less well prepared for coping with death (Zheng et al., 2021, 2022).

As suggested by other studies, such as the one by Wu et al., it is pivotal to address this issue in nursing students. They propose an alternative intervention methodology more focused on role-playing (Wu et al., 2023). While role-playing (Huang et al., 2023) and mindfulness-based training (Salem et al., 2025) have proven effective in enhancing empathy, emotional regulation, and resilience among nursing students (Huang et al., 2023; Salem et al., 2025), SBLE offers a high-fidelity, immersive environment where students receive real-time feedback, enabling the integration of technical and emotional skills in a dynamic, clinically relevant setting (Elendu et al., 2024). Compared to more traditional educational strategies, SBLE provides a more comprehensive preparation for complex care situations.

Although much of the early SBLE literature has primarily focused on the acquisition of technical competencies, such as procedural performance, medication safety, and clinical decision-making, there is a growing body of evidence supporting its application in non-technical domains. These include communication skills, emotional regulation, empathy development, and coping with emotionally complex clinical situations (Raghunathan et al., 2025). The present study aligns with this emerging line of research by focusing specifically on affective and relational competencies rather than purely technical outcomes.

In relation to burnout, our results are in line with those published by Andargeery et al. and Tlili et al. These authors perceive high levels of burnout in undergraduate students (Andargeery et al., 2025; Tlili et al., 2020). In our study, however, we go further, by demonstrating that there is a significant improvement in burnout scores after the students underwent the intervention. This result has important practical implications, as elevated burnout levels in nursing students may contribute to long-term psychological consequences, in line with evidence suggesting an association between burnout and future depression in nursing populations (Chen and Meier, 2021; Zhang et al., 2024).

In the realm of interventions aimed at reducing burnout, existing studies have predominantly concentrated on lowering stress and anxiety levels through various techniques such as relaxation methods (Cohen et al., 2023; Pelit-Aksu et al., 2021) and other therapeutic modalities (Tating et al., 2023). The current study introduces an intervention that targets burnout reduction from an academic perspective, emphasizing active learning methodologies.

Taken together, the improvements in CDS and burnout scores observed in our results may translate into enhanced emotional resilience, strengthened professional identity development, and more effective patient care under stressful conditions, particularly when working with terminally ill patients, as suggested by previous studies (Martín-Parrilla et al., 2025c; Martín-Parrilla, et al., 2025b). However, unlike these previous studies, which explored specific dimensions related to emotional preparedness and professional adaptation in nursing students, the present research integrates coping with death, burnout prevention, communication skills, and satisfaction with simulation-based learning within a single comprehensive educational intervention. In addition, this study incorporates both student self-perceptions and instructor-based assessments of communication competencies, thereby providing a broader and more integrative evaluation of the educational impact of SBLE in undergraduate nursing education.

### 4.3 Post intervention analysis

Regarding the results of satisfaction in high fidelity clinical simulation, it should be noted that undergraduate nursing students see clinical simulation as a useful tool for their learning. Another study with similar results to ours is that proposed by Alconero-Camarero et al. In this study carried out on a sample of nursing students, it is also showed very high general satisfaction with the SBLE sessions. They self-perceived that it helped them to learn from their own mistakes, and they highlighted the practical utility of the simulation process (Alconero-Camarero et al., 2020; Ruiz-Fernández et al., 2022).

Finally, due to the recent validation of the tool CSPR, there is a paucity of literature utilizing it in comparable studies. However, studies employing different instruments to assess communication skills in nursing students emphasize the imperative to incorporate such evaluations of non-technical competencies in this student demographic.

Previous studies have also focused their interventions on the development of non-technical skills, specifically communication skills, highlighting the importance of addressing this need through such interventions Chae et al. (2023) conducted an intervention study demonstrating that through simulation, specifically based on virtual reality, nursing students improved their communicative abilities. Öztürk et al., on the other hand, implemented a pedagogical model in their intervention centered on enhancing empathy (Öztürk et al., 2021; Ruiz-Fernández et al., 2022), a concept closely related to the innovative tool utilized in measuring the communicative abilities of our intervention.

The findings of these studies make it clear that there is a need to enhance nursing education in non-technical skills, particularly in the realm of communication. This necessitates the development of interventions and tools aligned with measuring how these skills facilitate the development of students before entering their careers as registered nurses. An illustrative example is the project undertaken by Leal-Costa et al. (2023), who devised an assessment tool for evaluating non-technical skills in nursing students, aiming to contribute to patient safety, a paradigm underpinning this research project.

One of the noteworthy outcomes of the study is the analysis conducted to ascertain whether there was a correlation between instructors’ perceptions of nursing students’ communication abilities during the briefing and debriefing phases, and students’ self-perceptions. Importantly, no previous studies have examined this specific correlation. As illustrated in Figures 3, 4, students’ self-perceived communication competencies aligned with their evaluations by instructors; those perceived to have stronger communication skills also received higher ratings. These findings underscore the importance of aligning perceived and actual communication skills in nursing education, ensuring accurate assessments and facilitating effective skill development.

Firstly, these findings suggest that students possess a relatively accurate self-awareness of their communication skills development during simulation-based training. This alignment supports the validity of self-assessment tools as complementary instruments alongside instructor evaluations, which can empower students to engage more actively in reflective learning processes.

Furthermore, the high correlation observed in the debriefing phase indicates that students not only recognize the educational value of debriefing but also that their perceived learning gains correspond closely with instructor assessments. This underscores the critical role of structured debriefing in fostering meaningful learning and skill acquisition in clinical communication. Educators might leverage these insights to reinforce the integration of self-assessment and instructor feedback in simulation curricula (Elendu et al., 2024), thereby enhancing learners’ metacognitive skills and promoting sustained improvements in communication competencies (Sanjaya et al., 2024).

In practical terms, promoting reflective self-assessment alongside instructor evaluation may enhance students’ ability to identify personal strengths and areas for improvement, thereby increasing their readiness for real-world clinical interactions with patients, families, and healthcare teams. This approach is consistent with discussions in previous research (Heydari and Beigzadeh, 2024). The findings support the continued use and refinement of dual-assessment strategies to maximize the educational impact of simulation-based communication training.

### 4.4 Comparison with recent simulation-based educational research

Recent quasi-experimental studies continue to support the educational value of SBLE in undergraduate nursing education, particularly in emotionally demanding clinical contexts. For example, recent evidence has shown that simulation-based interventions can significantly improve nursing students’ emotional preparedness, confidence, and communication abilities when facing end-of-life care situations and difficult clinical interactions (Marta, 2025; Son et al., 2025). Similarly, studies conducted in palliative and mental health education contexts have highlighted the usefulness of simulation methodologies for strengthening empathy, reflective capacity, and communication self-efficacy among nursing students (Alghamdi et al., 2024; Kim and Kim, 2024).

Other recent quasi-experimental interventions have focused on the relationship between simulation training and students’ emotional adaptation, stress management, and professional identity development. In this regard, SBLE has been associated with improvements in resilience, emotional regulation, and coping strategies during highly stressful clinical scenarios (Guo and Cui, 2026; Serafin et al., 2025). These studies reinforce the growing recognition of simulation not only as a technical training strategy but also as an educational methodology capable of supporting the emotional and psychological preparation of future healthcare professionals.

However, unlike previous studies, which predominantly examined isolated educational outcomes such as communication, emotional preparedness, or self-efficacy independently, the present study integrates coping with death, burnout prevention, communication skills, and satisfaction with simulation-based learning within a single comprehensive educational intervention. In addition, this research combines students’ self-perceptions with instructor-based assessments of communication competencies, thereby providing a broader and more integrative evaluation of the educational impact of SBLE in undergraduate nursing education and end-of-life care training contexts.

### 4.5 Study limitations

This study was designed as a program evaluation without a control group, which limits causal and comparative interpretations. Specifically, it was not possible to compare the SBLE intervention with alternative teaching strategies or with a non-intervention condition, which restricts the ability to attribute observed changes exclusively to the intervention. This limitation is consistent with classical discussions on quasi-experimental designs in educational research (Campbell and Stanley, 1963) and remains relevant in contemporary simulation-based learning studies, where non-randomized designs are still widely used due to curricular and ethical constraints in healthcare education (Sohrabi et al., 2025). However, the goal was to examine the implementation, feasibility, and potential educational value of the program in a real-world academic context.

The use of convenience sampling facilitated participant recruitment and enabled the integration of the intervention into existing curricular structures. While this approach may limit the generalizability of the findings and introduce selection bias, it remains a commonly used strategy in nursing education research where access to defined student cohorts is required (Jager et al., 2017). Recent studies continue to report similar sampling approaches in simulation-based educational interventions, acknowledging both feasibility advantages and limitations in external validity (Jackson et al., 2024).

Beyond the overall design constraints, the analysis focused on global scale scores rather than item-level responses, which may have limited the granularity of the findings. In particular, item-specific analysis of emotionally salient statements could have provided additional insight into students’ emotional processing and adaptation throughout the intervention. This limitation is consistent with psychometric literature indicating that global scoring enhances reliability but reduces sensitivity to item-level variation (McClure et al., 2024).

The absence of a dismantling design limits the ability to determine whether the observed improvements were primarily driven by the pre-briefing, the simulation experience itself, or the debriefing phase. This restricts causal attribution to specific components of the SBLE intervention. This limitation has been widely discussed in simulation pedagogy literature, where the interdependence of simulation phases is recognized as a methodological challenge (Jackson et al., 2024; Oliveira Silva et al., 2023; Salifu et al., 2022). Recent evidence continues to emphasize the difficulty of isolating active ingredients in simulation-based interventions (Salifu et al., 2022).

Additionally, the study relied on self-reported data, which may be subject to social desirability bias and limited introspective accuracy. Although efforts were made to triangulate data using both self-perception and instructor-rated instruments, these sources remain subjective. This limitation is well established in psychological and educational measurement theory (Rosenman et al., 2011), and recent studies continue to confirm its relevance in nursing education research involving emotional constructs and simulation-based learning outcomes (Ahn et al., 2023).

Moreover, the short follow-up period limits our ability to determine the persistence of observed effects over time. Longitudinal designs are recommended in educational intervention research to assess durability of learning outcomes (Hart et al., 2024), and recent simulation-based education studies confirm that most interventions still rely on immediate post-test designs, limiting evidence on long-term retention (Alharbi et al., 2024; Legoux et al., 2021).

From a conceptual perspective, it is important to acknowledge that fear of death is a complex and multifaceted phenomenon influenced by numerous extraneous factors such as age, religious beliefs, cultural background, and previous personal experiences. The cross-cultural context also plays a significant role in shaping individuals’ perceptions and coping mechanisms related to death anxiety. While the present study focused primarily on assessing the presence and impact of fear of death within the sample, it did not delve into the exploration of these underlying influences. This limitation is consistent with existential psychology frameworks (Rezapour, 2022).

Future studies should aim to enhance both internal and external validity by employing randomized controlled trials and probabilistic sampling methods. Incorporating objective or behavioral measures would provide a valuable complement to self-report data and reduce potential biases. Additionally, longitudinal research is recommended to evaluate the long-term effects of simulation-based interventions on communication, emotional resilience, and coping with death. Finally, exploring the influence of individual and cultural factors on fear of death would contribute to a more nuanced understanding of this phenomenon and its implications for nursing education and professional practice.

## 5 Conclusion

Our hypothesis was that participation in the comprehensive SBLE would improve nursing students’ coping with death and communication skills, reduce burnout levels, and generate high levels of satisfaction with the educational experience. The findings of this study support this hypothesis, as the implementation of SBLE not only enhanced undergraduate nursing students’ coping with death and reduced burnout levels but also demonstrated a highly positive and engaging educational experience. Moreover, the results demonstrate the successful cultivation of effective communication skills among students.

These outcomes suggest that SBLE can be effectively integrated into nursing curricula to better prepare students for the emotional and communicative challenges they will face in clinical practice, particularly in end-of-life care settings.

By fostering coping skills, reducing burnout, and improving communication competencies, SBLE has the potential to contribute to improved patient care quality and safety. Educational institutions and healthcare educators should consider adopting simulation-based learning as a core component of training programs to enhance student readiness and professional development.

## Data Availability

The raw data supporting the conclusions of this article will be made available by the authors, without undue reservation.
